# Association between MTR A2756G polymorphism and susceptibility to congenital heart disease: A meta-analysis

**DOI:** 10.1371/journal.pone.0270828

**Published:** 2022-07-08

**Authors:** Wanru Liu, Jing Wang, Lin-jiao Chen

**Affiliations:** Center for Reproductive Medicine, Center for Prenatal Genetics, First Hospital of Jilin University, Changchun, Jilin, China; Texas A&M University College Station, UNITED STATES

## Abstract

The association between methionine synthase (MTR) A2756G (rs1805087) polymorphism and the susceptibility to congenital heart disease (CHD) has not been fully determined. A meta-analysis of case-control studies was performed to systematically evaluate the above association. Studies were identified by searching the PubMed, Embase, Web of Science, China National Knowledge Infrastructure, and WanFang databases from inception to June 20, 2021. Two authors independently performed literature search, data extraction, and quality assessment. Predefined subgroup analyses were carried out to evaluate the impact of the population ethnicity, source of healthy controls (community or hospital-based), and methods used for genotyping on the outcomes. A random-effects model was used to combine the results, and 12 studies were included. Results showed that MTR A2756G polymorphism was not associated with CHD susceptibility under the allele model (odds ratio [OR]: 0.96, 95% confidence interval [CI]: 0.86 to 1.07, P = 0.43, I^2^ = 4%), heterozygote model (OR: 0.95, 95% CI: 0.84 to 1.07, P = 0.41, I^2^ = 0%), homozygote model (OR: 1.00, 95% CI: 0.64 to 1.55, P = 0.99, I^2^ = 17%), dominant genetic model (OR: 0.95, 95% CI: 0.84 to 1.07, P = 0.41, I^2^ = 0%), or recessive genetic model (OR: 0.94, 95% CI: 0.62 to 1.43, P = 0.32, I^2^ = 13%). Consistent results were found in subgroup analyses between Asian and Caucasian populations in studies with community and hospital-derived controls as well as in studies with PCR-RFLP and direct sequencing (all P values for subgroup differences > 0.05). In conclusion, current evidence does not support an association between MTR A2756G polymorphism and CHD susceptibility.

## Introduction

Congenital heart disease (CHD) is a common birth defect in newborns that has been associated with increased morbidity and mortality for infants [1, 2]. Accumulating evidence from clinical studies suggests that folic acid deficiency in women during pregnancy is associated with a higher risk of CHD for the fetus [3]. Folic acid supplementation has been associated with a series of birth defects, such as neural tube defects and CHD [4]. Moreover, genetic studies also showed that the genotype status of genes that play important roles in the metabolic pathway for folic acid may affect the risk of CHD [5–7]. Previous studies confirmed that two classical variants of 5,10-methylenetetrahydrofolate reductase (MTHFR), namely MTHFR C677T (rs1801133) and A1298C polymorphisms, are associated with an increased risk of CHD [8, 9]. Previous association studies were focused on the influence of the methionine synthase (MTR) variant on CHD susceptibility [10–12]. Physiologically, MTR is a key enzyme involved in the metabolism of folic acid and catalyzes the remethylation of homocysteine to methionine during the removal of homocysteine [13, 14]. The MTR A2756G (rs1805087) polymorphism, a classical genetic variant, can lead to the deletion mutation of codon D919G and therefore affect the enzyme activity of MTR [13, 14]. A growing number of studies have been performed to evaluate the association between MTR A2756G polymorphism and CHD susceptibility [15–26]. However, a conclusion remains to be determined. Therefore, we performed a meta-analysis of case-control studies to summarize the potential associations between MTR A2756G polymorphism and CHD susceptibility. The possible influences of the study characteristics, such as the ethnicity of the participants, source of healthy controls, and methods used for genotyping, on the association were also explored in subgroup analyses.

## Materials and methods

The Meta-analysis of Observational Studies in Epidemiology [27] Statement and Cochrane’s Handbook [28] were followed for the design, performance, and reporting of this meta-analysis. The protocols and analytical strategies for the meta-analysis were also in accordance with those for previous meta-analyses of studies related to SNPs [29–32].

### Search strategy

Electronic databases including PubMed, Embase, Web of Science, China National Knowledge Infrastructure, and WanFang were searched with a combination of the following terms: (1) "MTR" OR "Methionine synthase" OR "A2756G" OR "rs1805087"; (2) "Heart Defects, Congenital" OR "congenital heart abnormalities" OR "congenital heart abnormality" OR "congenital heart malformation" OR "congenital heart defect" OR "congenital heart disease" OR "congenital heart defects" OR "congenital heart diseases" OR "congenital anomalies" OR "birth defect"; and (3) "Polymorphism, Single Nucleotide" OR "Genotype" OR "Alleles" OR "polymorphism" OR "genetic variant" OR "genetic variants" OR "genetic polymorphism" OR "genetic" OR "Genetic Variation" OR "SNP" OR "mutation" OR "variation" OR "variant" OR "single nucleotide polymorphism". Studies published in English or Chinese were considered. The reference lists of related original and review articles were manually searched for potentially eligible studies. The final literature search was performed on June 20, 2021.

### Study selection

Studies fulfilling all of the following criteria were included: (1) case-control studies published as full-length articles; (2) included patients with confirmed CHD diagnosis and healthy participants as controls; (3) MTR A2756G polymorphism was evaluated and regarded as exposure; and (4) reported the association between the MTR A2756G polymorphism status and susceptibility to CHD. Reviews, editorials, studies reporting the maternal genotype of the patients and controls, studies without healthy controls, and studies without detailed genotype data were excluded from the current meta-analysis.

### Data extraction and quality evaluation

The literature search, data extraction, and quality assessment of the included studies were performed by two authors independently according to the predefined criteria. Discrepancies were resolved by consensus. The extracted data were as follows: (1) name of the first author, publication year, and country; (2) participant characteristics, including the ethnicity of the population and source of healthy controls; (3) genotyping methods; and (4) distributions of participants with MTR A2756G genotype status (AA, AG, and GG). The quality of each study was evaluated using the Newcastle-Ottawa Scale (NOS) [33]. This scale ranges from 1 to 9 and judges the quality of case-control studies according to the selection of the study groups, comparability of the groups, and ascertainment of exposure.

### Statistical analyses

For each study, the Hardy-Weinberg equilibrium (HWE) was tested to examine possible biases in genotype distribution. Odds ratios (ORs) and their corresponding 95% confidence intervals (CIs) were applied as the general measure for the association between the MTR A2756G genotype status and CHD susceptibility. The pooled ORs and 95% CIs were calculated for five genetic models: allele model (G versus A), heterozygote model (AG versus AA), homozygote model (GG versus AA), dominant model (GG + AG versus AA), and recessive model (GG versus AG + AA). Cochrane’s Q test was used to evaluate the heterogeneity among the included cohort studies as well as to estimate the I^2^ statistic [34]. I^2^ > 50% indicated significant heterogeneity. A random-effects model was used to synthesize the hazard ratio (HR) data because this model is considered a more generalized method that incorporates potential heterogeneity among the included studies [28]. Sensitivity analysis, conducted by excluding one study at a time, was performed to test the stability of the results [35]. Predefined subgroup analyses were carried out to evaluate the impact of the population ethnicity, source of healthy controls (community or hospital-based), and methods used for genotyping on the outcomes. Publication bias was assessed by visual inspection of the funnel plots for symmetry as well as by Egger’s regression asymmetry test [36]. RevMan (Version 5.1; Cochrane Collaboration, Oxford, UK) software was used to perform the meta-analysis and statistical analysis.

## Results

### Literature search

**Fig 1:**
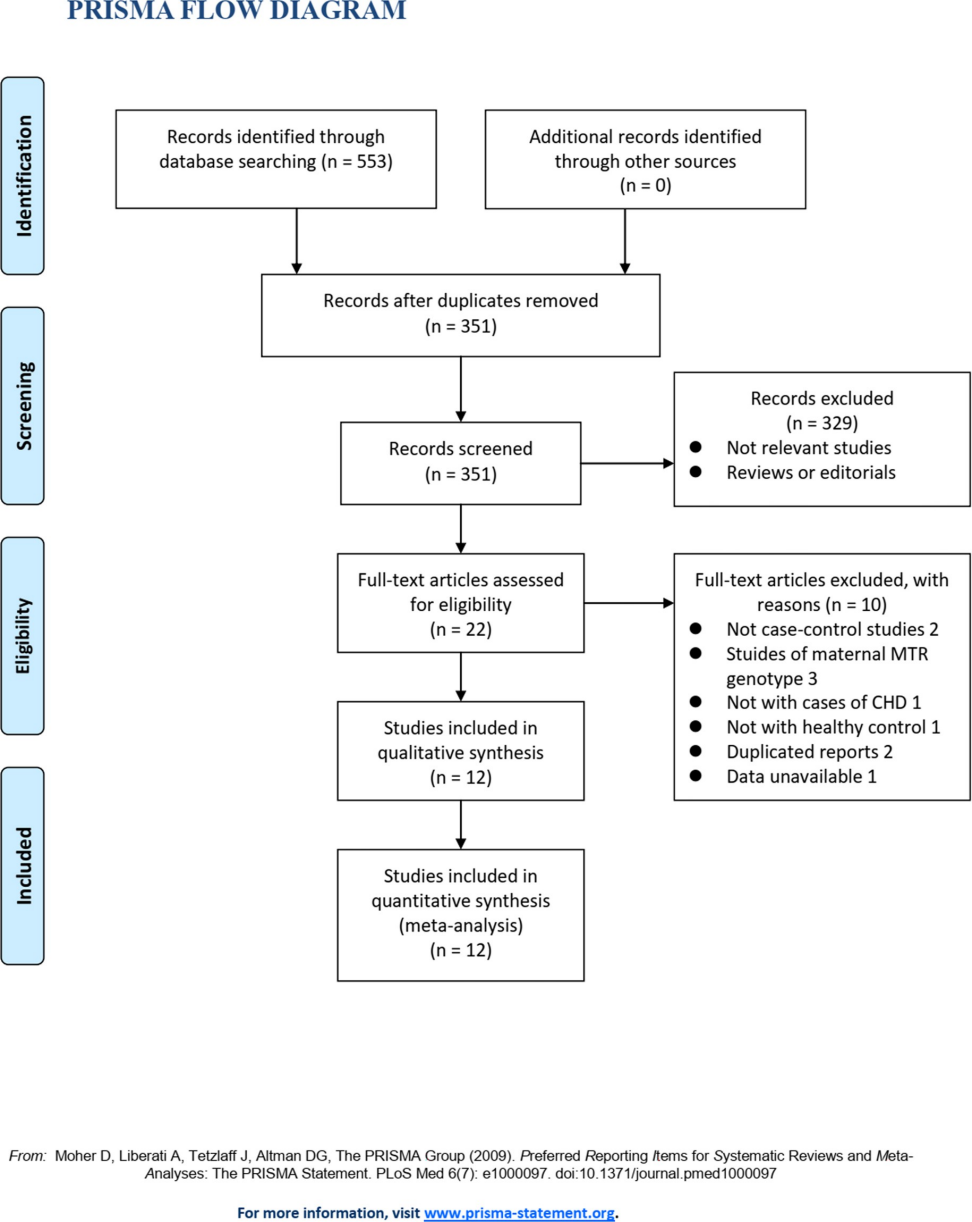
Flowchart of the database search and study identification.

**Fig 1** shows the process for the database search. Briefly, 351 articles were obtained during the initial literature search of the databases after excluding duplicates. Among them, 329 articles were excluded for lack of relevance after screening the titles and abstracts. Subsequently, 22 articles underwent full-text review. Of these, 10 articles were further excluded for the reasons listed in **Fig 1**. Finally, 12 studies were obtained for this meta-analysis [15–26].

### Study characteristics and quality evaluation

The characteristics of the included studies are summarized in **Table 1**. One article included three comparisons from different centers in China; therefore, the datasets were included in the meta-analysis separately [22]. Overall, 12 case-control studies including 3853 patients with CHD and 3776 healthy controls were obtained for the meta-analysis. These studies were published between 2004 and 2018, and were performed in China, India, Malaysia, Brazil, and the United States, separately. Most of the studies included community-based healthy controls, with the exception of two studies that included healthy controls recruited in a hospital setting who visited the clinics for health examinations [16, 19]. Regarding the methods used for genotyping, polymerase chain reaction followed by restriction fragment length polymorphism (PCR-RFLP) analyses were performed in six studies [15–17, 19, 21, 24], and direct sequencing was applied in the remaining studies [18, 20, 22, 23, 25, 26]. The sample size for the included studies ranged from 64 to 2049 and the distributions of the MTR A2756G genotype status for all of the included studies were in agreement with HWE (P all > 0.05). The NOS scores were 7~9 for all of the included studies, indicating good study quality (**Table 2**).

**Table 1:** Characteristics and genotype status of MTR A2756G in patients and controls for the included studies.

Study	Country	Ethnicity	Source of control	Genotyping method	Patients				Controls				P for HWE
					AA	AG	GG	Total	AA	AG	GG	Total	
Zhu 2004	China	Asian	Community-based	PCR-RFLP	169	17	0	186	92	11	0	103	0.567
Galdieri 2007	Brazil	Caucasian	Hospital-based	PCR-RFLP	36	20	2	58	22	13	3	38	0.588
Liu 2007	China	Asian	Community-based	PCR-RFLP	120	12	0	132	97	10	0	107	0.612
Shaw 2009	USA	Caucasian	Community-based	Direct sequencing	141	66	7	214	144	69	7	220	0.715
Gong 2010	China	Asian	Hospital-based	PCR-RFLP	48	12	0	60	43	17	0	60	0.201
Wang 2013	China	Asian	Community-based	Direct sequencing	132	27	1	160	153	33	2	188	0.883
Zhao 2014a	China	Asian	Community-based	Direct sequencing	513	80	9	602	567	87	6	660	0.251
Zhao 2014b	China	Asian	Community-based	Direct sequencing	627	103	5	735	459	97	8	564	0.998
Zhao 2014c	China	Asian	Community-based	Direct sequencing	891	104	8	1003	913	129	4	1046	0.252
Mohamad 2014	Malaysia	Asian	Community-based	PCR-RFLP	106	41	3	150	114	36	0	150	0.104
Shi 2015	China	Asian	Community-based	Direct sequencing	107	31	0	138	174	33	0	207	0.212
Elizabeth 2017	India	Asian	Community-based	PCR-RFLP	11	14	7	32	18	9	5	32	0.078
Su 2018	China	Asian	Community-based	Direct sequencing	87	82	14	183	96	78	27	201	0.088
Duan 2018	China	Asian	Community-based	Direct sequencing	164	32	4	200	160	37	3	200	0.612

MTR, methionine synthase; PCR-RFLP: polymerase chain reaction followed by restriction fragment length polymorphism; HWE, Hardy-Weinberg equilibrium.

**Table 2:** Quality evaluation for the included case-control studies via the Newcastle-Ottawa Scale.

Study	Adequate definition of cases	Representativeness of cases	Selection of control	Definition of control	Control for age and sex	Control for other factors	Ascertainment of exposure	Same method of ascertainment for case and control	None response rate	Total
Zhu 2004	1	1	1	1	1	0	1	1	1	8
Galdieri 2007	1	1	0	1	1	0	1	1	1	7
Liu 2007	1	1	1	1	1	0	1	1	1	8
Shaw 2009	1	1	1	1	1	0	1	1	1	8
Gong 2010	1	1	0	1	1	0	1	1	1	7
Wang 2013	1	1	1	1	1	0	1	1	1	8
Zhao 2014	1	1	1	1	1	1	1	1	1	9
Mohamad 2014	1	1	1	1	0	0	1	1	1	7
Shi 2015	1	1	1	1	0	0	1	1	1	7
Elizabeth 2017	1	1	1	1	1	0	1	1	1	8
Su 2018	1	1	1	1	1	0	1	1	1	8
Duan 2018	1	1	1	1	0	0	1	1	1	7

## MTR A2756G polymorphism and CHD susceptibility

**Fig 2:**
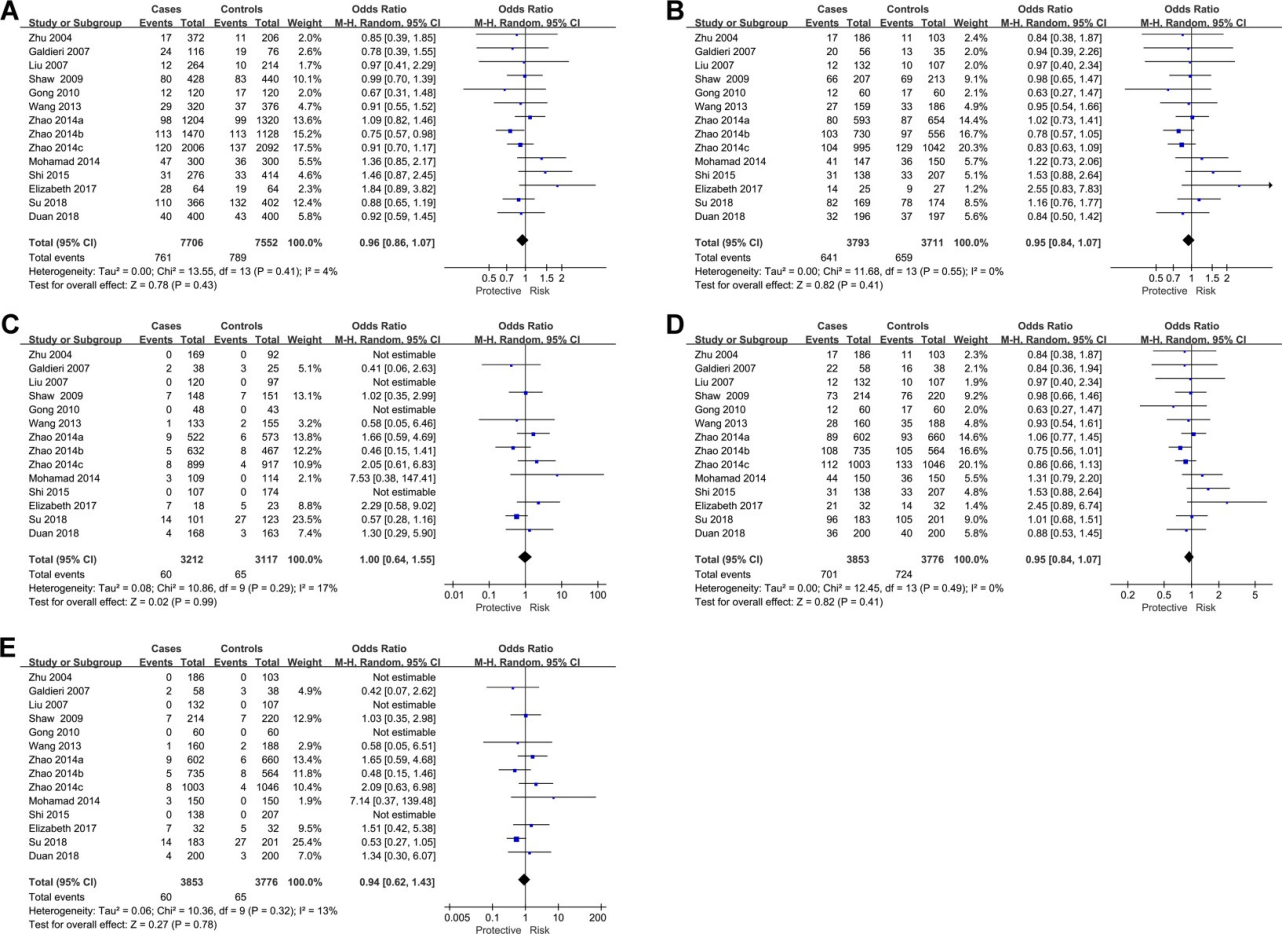
Forest plots for the meta-analysis of the association between MTR A2756G polymorphism and CHD susceptibility in different genetic models. A, meta-analysis under the allele model; B, meta-analysis under the heterozygote model; C, meta-analysis under the homozygote model; D, meta-analysis under the dominant genetic model; and E, meta-analysis under the recessive genetic model.

Pooled results from the 14 datasets on 12 case-control studies showed that MTR A2756G polymorphism was not associated with CHD susceptibility under the allele model (OR: 0.96, 95% CI: 0.86 to 1.07, P = 0.43, I^2^ = 4%; **Fig 2A**), heterozygote model (OR: 0.95, 95% CI: 0.84 to 1.07, P = 0.41, I^2^ = 0%; **Fig 2B**), homozygote model (OR: 1.00, 95% CI: 0.64 to 1.55, P = 0.99, I^2^ = 17%; **Fig 2C**), dominant genetic model (OR: 0.95, 95% CI: 0.84 to 1.07, P = 0.41, I^2^ = 0%; **Fig 2D**), or recessive genetic model (OR: 0.94, 95% CI: 0.62 to 1.43, P = 0.32, I^2^ = 13%; **Fig 2E**). No significant heterogeneity was observed for the above meta-analyses (P for Cochrane’s Q test = 0.41, 0.55, 0.29, 0.49, and 0.32, respectively). Further sensitivity analyses by excluding one dataset at a time showed consistent results (ORs under allele model: 0.93~1.00, ORs under heterozygote model: 0.93~0.99, ORs under homozygote model: 0.90~1.21, ORs under dominant genetic model: 0.93~1.00, ORs under recessive genetic model: 0.85~1.17; all P > 0.05). Consistent results were found in subgroup analyses between Asian and Caucasian populations in studies with community and hospital-derived controls as well as in studies with PCR-RFLP and direct sequencing (**Table 3**; all P values for subgroup differences > 0.05).

**Table 3:** Subgroup analysis for the association between MTR A2756G and CHD susceptibility.

	Datasets	OR (95% CI)	P for subgroup effect	I^2^	P for subgroup difference
Allele					
Ethnicity					
Asian	12	0.97 [0.85, 1.10]	0.62	17%	
Caucasian	2	0.94 [0.70, 1.28]	0.71	0%	0.89
Source of control					
Community-based	12	0.97 [0.86, 1.10]	0.66	12%	
Hospital-based	2	0.73 [0.44, 1.23]	0.24	0%	0.30
Genotyping method					
PCR-RFLP	6	1.07 [0.80, 1.45]	0.64	12%	
Direct sequencing	8	0.93 [0.83, 1.05]	0.24	0%	0.38
Heterozygote					
Ethnicity					
Asian	12	0.95 [0.83, 1.09]	0.49	6%	
Caucasian	2	0.97 [0.67, 1.41]	0.87	0%	0.93
Source of control					
Community-based	12	0.96 [0.84, 1.09]	0.51	0%	
Hospital-based	2	0.77 [0.42, 1.41]	0.39	0%	0.48
Genotyping method					
PCR-RFLP	6	1.05 [0.76, 1.44]	0.78	0%	
Direct sequencing	8	0.93 [0.82, 1.07]	0.31	0%	0.51
Homozygote					
Ethnicity					
Asian	12	1.09 [0.63, 1.87]	0.76	30%	
Caucasian	2	0.81 [0.32, 2.06]	0.66	0%	0.60
Source of control					
Community-based	12	1.05 [0.66, 1.66]	0.83	20%	
Hospital-based	2	0.41 [0.06, 2.63]	0.35	NA	0.33
Genotyping method					
PCR-RFLP	6	1.57 [0.37, 6.73]	0.54	41%	
Direct sequencing	8	0.90 [0.58, 1.39]	0.62	8%	0.47
Dominant					
Ethnicity					
Asian	12	0.96 [0.83, 1.10]	0.57	11%	
Caucasian	2	0.95 [0.67, 1.36]	0.79	0%	0.97
Source of control					
Community-based	12	0.97 [0.85, 1.10]	0.59	4%	
Hospital-based	2	0.73 [0.40, 1.32]	0.30	0%	0.37
Genotyping method					
PCR-RFLP	6	1.06 [0.77, 1.47]	0.71	8%	
Direct sequencing	8	0.93 [0.82, 1.06]	0.29	0%	0.46
Recessive					
Ethnicity					
Asian	12	1.02 [0.61, 1.71]	0.94	27%	
Caucasian	2	0.82 [0.33, 2.06]	0.67	0%	0.69
Source of control					
Community-based	12	0.99 [0.64, 1.54]	0.97	17%	
Hospital-based	2	0.42 [0.07, 2.62]	0.35	NA	0.37
Genotyping method					
PCR-RFLP	6	1.26 [0.36, 4.45]	0.72	29%	
Direct sequencing	8	0.89 [0.57, 1.42]	0.64	16%	0.62

MTR, methionine synthase; CHD, congenital heart disease; OR, odds ratio; CI, confidence interval; PCR-RFLP: polymerase chain reaction followed by restriction fragment length polymorphism; NA, not applicable.

### Publication bias

**Fig 3:**
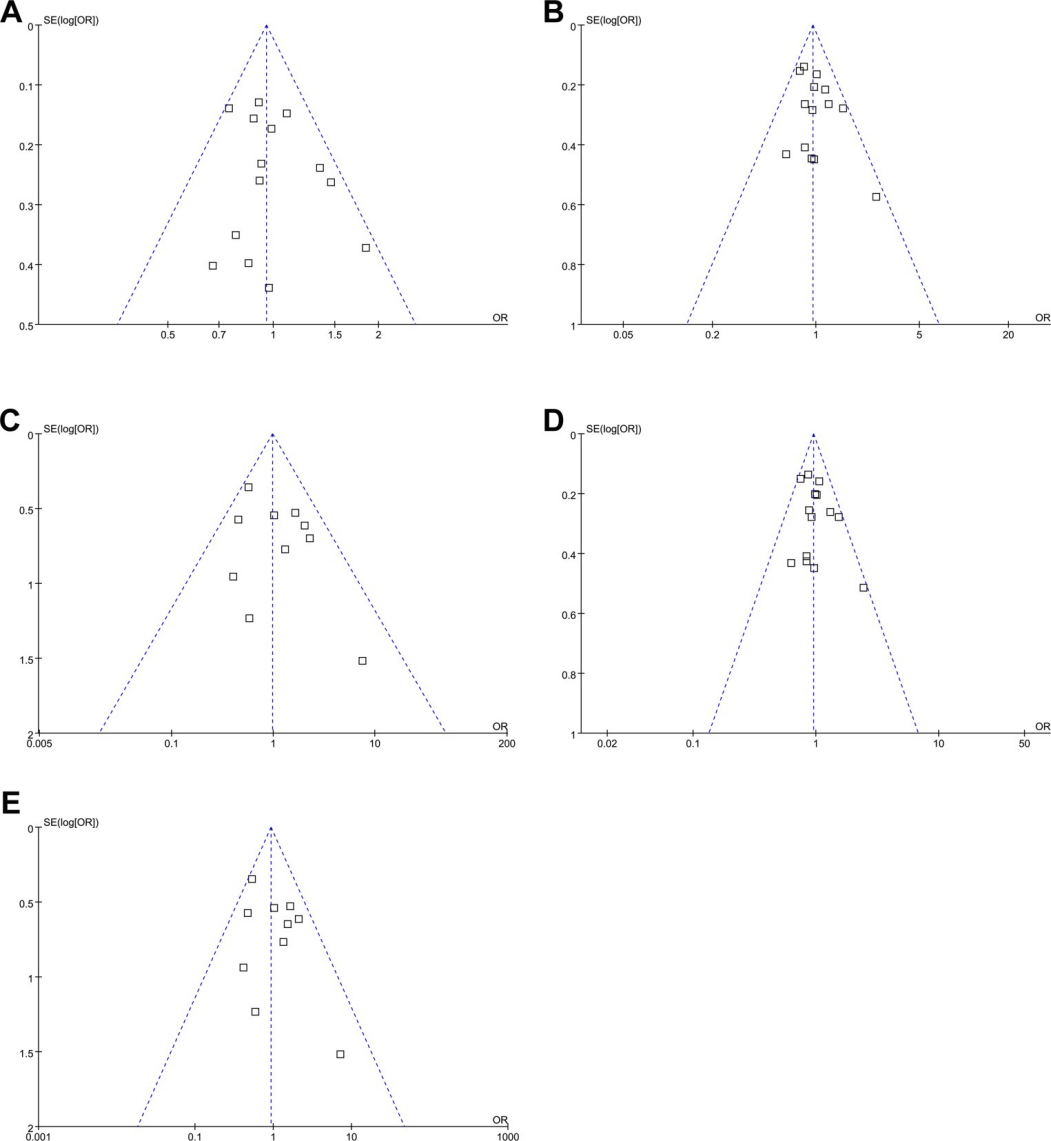
Funnel plots for the publication bias underlying the meta-analysis of the association between MTR A2756G polymorphism and CHD susceptibility in different genetic models. A, funnel plots for the meta-analysis under the allele model; B, funnel plots for the meta-analysis under the heterozygote model; C, funnel plots for the meta-analysis under the homozygote model; D, funnel plots for the meta-analysis under the dominant genetic model; and E, funnel plots for the meta-analysis under the recessive genetic model.

Funnel plots for the association between MTR A2756G polymorphism and susceptibility to CHD in different models are shown in **Fig 3A to 3E.** The funnel plots were symmetrical on visual inspection, suggesting a low risk of publication bias. The results of Egger’s regression tests also did not indicate significant publication bias underlying the meta-analyses (all P > 0.10).

## Discussion and conclusion

In this meta-analysis of case-control studies, we found that the MTR A2756G polymorphism is not significantly associated with CHD susceptibility. The reliability of the findings was evidenced by the consistency of the results for five genetic models, as well as the results of sensitivity and subgroup analyses. Specifically, sensitivity analysis by excluding one study at a time showed that the results were not primarily driven by any of the included studies. Subgroup analyses showed that there was no significant influence from the predefined study characteristics on the association, including the ethnicity of the participants, source of healthy controls, and genotyping methods. Taken together, the current evidence from case-control studies does not support a significant association between MTR A2756G polymorphism and CHD susceptibility.

To the best of our knowledge, only one previous meta-analysis evaluated the potential association between the MTR A2756G polymorphism and CHD susceptibility [10]. The previous meta-analysis included four case-control studies published before 2014 (482 participants) and showed that A2756G in MTR was not significantly associated with susceptibility to CHD [10]. Although the results of the previous meta-analysis were consistent with those of the current study, the limited number of datasets and small sample size included in the previous meta-analysis may lead to an inadequate statistical power for the detection of a potentially significant association between MTR A2756G polymorphism and CHD susceptibility. Moreover, only one genetic model was applied (allele) and subgroup analyses could not be performed in the previous study [10]. Our meta-analysis, on the other hand, was performed with 7629 participants following an intensive literature search and the inclusion of up-to-date studies. The remarkably larger sample size guaranteed adequate statistical power and the feasibility of multiple sensitivity and subgroup analyses. Collectively, the results of the main meta-analysis with five genetic models and subgroup analyses in our study consistently showed that MTR A2756G polymorphism was not significantly associated with CHD susceptibility. This is consistent with the previous meta-analyses that evaluated the association between MTR A2756G polymorphism and folic acid deficiency-related birth defects. Early meta-analyses including 10–13 studies showed that MTR A2756G polymorphism is not associated with neural tube defect risk, and the results were consistent in the overall and Caucasian populations [37–39]. Similarly, a meta-analysis of nine studies also showed that maternal gene polymorphism of MTR A2756G did not significantly affect the risk of Down syndrome in the offspring [40]. Additionally, a recent meta-analysis of 12 case-control studies indicated that MTR A2756G polymorphism may not be associated with the risk of nonsyndromic cleft lip with or without a cleft palate (NSCL/P) [41]. These findings may reflect the complexity of environmental and genetic interactions during the pathogenesis of birth defects. A previous study suggested a significant gene–gene interaction between the MTR A2756G polymorphism and MTHFR (rs1801133) in determining the susceptibility to NSCL/P, which was not significant if only MTR A2756G polymorphism was considered [42]. Similarly, a recent study showed that a haplotype CAA (rs1770449-rs1805087-rs1050993) in MTR rather than MTR A2756G alone was associated with the total CHD risk [43]. Future studies that incorporate gene–gene and gene–environment interactions are needed to determine the role of certain variants in the pathogenesis of CHD.

Although the cause of the majority of CHD cases is unknown, advances in genetic CHD studies provide increasing evidence for genetic causes underlying CHD and have identified critical biological pathways involved in CHD, including chromatin remodeling, Notch signaling, cilia function, RAS signaling, and gut immunodeficiency etc. [5, 44–47]. In the present manuscript, we evaluated the correlation between MTR A2756G and susceptibility to CHD by meta-analysis based on 12 studies. Although only one SNP (MTR A2756G) was investigated, the role of MTR in the development of human diseases is critical because it has been a hot spot in recent genetic studies. MTR A2756G is a common nonsynonymous polymorphism in the gene that encodes MTR, a key enzyme in the pathway leading to DNA methylation that catalyzes the remethylation of homocysteine to methionine [48]. It has been reported that MTR A2756G increases the risk of cancer, such as pediatric acute lymphoblastic leukemia [49], prostate cancer [50], breast cancer etc. [51]. MTR A2756G is also involved in the regulation of folate metabolism, which is profoundly implicated in the DNA methylation pathway. Multiple maternal factors are thought to contribute to CHD development, including folate intake [4]. Maternal DNA methylation, which is dependent on folate metabolism, may also impact the risk of CHDs. For example, there was a report that revealed the association between maternal DNA methylation and CHD risk [52]. Taken together, considering the important role of MTR A2756G in the biological process, we believe that a meta-analysis to evaluate the correlation between MTR A2756G and susceptibility to CHD is reasonable and meaningful. Although we used an extensive search strategy in this study, almost all studies aiming to evaluate the SNPs of MTR and CHD focused on the MTR A2756G polymorphism. Only one study included other SNPs of MTR, which showed that two regulatory variants of MTR, -186T>G and +905G>A, were associated with an increased risk of CHD [22]. Due to the limited datasets available for other SNPs of MTR, a meta-analysis was not performed for other variants of the MTR gene. We also acknowledge the necessity and value of multi-gene SNPs or multi-SNPs of the same gene for their associations with CHD, and future studies are warranted.

The meta-analysis conducted in this study was based on association studies only. Despite significant progress in dissecting the genetic architecture of complex diseases by genome-wide association studies (GWAS), the genetic variants identified by GWAS can only explain a small proportion of the heritability of complex diseases. Association analysis (including SNP studies) is a major tool for genomic studies of complex diseases and has been used for decades. Although novel technologies have been developed to uncover hidden genetic variants, association analysis still lacks the power to determine the mechanisms of diseases due to its inability to identify causal signals, which are quite different from association signals. Another reason is that the widespread networks established from integrated omics analysis are undirected. Because association analysis has limited power to unravel the mechanisms of complex diseases, it is necessary to shift the paradigm of genomic analysis from association analysis to causal inference analysis [53]. Causal inference is the process of determining the independent, actual effect of a particular phenomenon that is a component of a larger system [54]. Causal inference analysis includes several algorithms, such as intervention, domain shift learning, temporal structure, and counterfactual thinking, all of which are used as major concepts to understand causation and reasoning. Moreover, Mendelian randomization (MR) is an analytic technique that uses genetic variants as instrumental variables to test for the causative association between an exposure and an outcome [55]. Therefore, bidirectional MR analysis is becoming increasingly efficient and cost-effective (with strong power) for analyzing GWAS data with multiple genetic variants [55]. In this regard, future studies with causal inference analysis or Mendelian randomization analysis are warranted to address the possible causative associations between MTR A2756G and CHD.

Artificial intelligence (AI) and machine learning (ML) have recently received enormous attention due to the successful application of deep neural networks in many fields, including medical science [55, 56]. However, the essential components of causal inference analysis are often overlooked by ML, leading to some failures in deep learning. This suggests that AI coupling with causal inference analysis is still under-developed in the current stage; for example, deep learning for nonlinear mediation and instrumental variable causal analysis or the construction of causal networks as a continuous optimization problem. Future studies evaluating the feasibility of genetic polymorphism-based ML models for predicting the risk of CHD may also be performed.

Our study also has limitations. Firstly, eight of the included studies were performed in China and the results were mostly from Chinese populations. Only two studies included Caucasian populations; therefore, the results should be validated in large-scale studies. The association between MTR A2756G polymorphism and CHD susceptibility in other ethnic groups such as Africans should be investigated in the future. Secondly, outcomes according to the individual forms of CHD were rarely reported among the included studies. Therefore, we were unable to evaluate the possible associations between MTR A2756G polymorphism and individual forms of CHD. Future studies are warranted. In addition, this is a meta-analysis based on study-level data rather than data for individual patients. Therefore, the influence of age, sex, and other characteristics on the association between MTR A2756G polymorphism and susceptibility to CHD remains unknown. Finally, the results were based on estimates with univariate analysis. An imbalance in participant characteristics between the patients and controls may confound the results.

In conclusion, the results of this meta-analysis indicated that current evidence from case-control studies does not support an association between MTR A2756G polymorphism and CHD susceptibility.

## Abbreviations

CHD: congenital heart disease
MTHFR: 5,10-methylenetetrahydrofolate reductase
MTR: methionine synthase
NOS: Newcastle-Ottawa Scale
HWE: Hardy-Weinberg equilibrium
HR: hazard ratio

## References

[1] Gaur L, Cedars A, Diller GP, Kutty S, Orwat S. Management considerations in the adult with surgically modified d-transposition of the great arteries. Heart. 2021. Epub 2021/03/21. heartjnl-2020-318833 [pii] doi: 10.1136/heartjnl-2020-318833 . 33741578

[2] Zhang TN, Wu QJ, Liu YS, Lv JL, Sun H, Chang Q, et al. Environmental Risk Factors and Congenital Heart Disease: An Umbrella Review of 165 Systematic Reviews and Meta-Analyses With More Than 120 Million Participants. Front Cardiovasc Med. 2021;8:640729. Epub 2021/04/02. doi: 10.3389/fcvm.2021.640729 ; PubMed Central PMCID: PMC8006458. 33791351 PMC8006458

[3] Czeizel AE, Dudas I, Vereczkey A, Banhidy F. Folate deficiency and folic acid supplementation: the prevention of neural-tube defects and congenital heart defects. Nutrients. 2013;5(11):4760–75. Epub 2013/11/29. doi: 10.3390/nu5114760 [pii]. ; PubMed Central PMCID: PMC3847759. 24284617 PMC3847759

[4] Soheilirad Z. Folic acid intake in prevention of congenital heart defects: A mini evidence review. Clin Nutr ESPEN. 2020;38:277–9. Epub 2020/07/22. S2405-4577(20)30111-X [pii] doi: 10.1016/j.clnesp.2020.05.021 . 32690169

[5] Pierpont ME, Brueckner M, Chung WK, Garg V, Lacro RV, McGuire AL, et al. Genetic Basis for Congenital Heart Disease: Revisited: A Scientific Statement From the American Heart Association. Circulation. 2018;138(21):e653–e711. Epub 2018/12/21. doi: 10.1161/CIR.0000000000000606 ; PubMed Central PMCID: PMC6555769. 30571578 PMC6555769

[6] Wang BJ, Chen Y. [Folic acid metabolism gene polymorphism and congenital heart disease]. Zhonghua Er Ke Za Zhi. 2012;50(8):630–3. Epub 2012/11/20. . 23158745

[7] Coppede F. The genetics of folate metabolism and maternal risk of birth of a child with Down syndrome and associated congenital heart defects. Front Genet. 2015;6:223. Epub 2015/07/15. doi: 10.3389/fgene.2015.00223 ; PubMed Central PMCID: PMC4479818. 26161087 PMC4479818

[8] Yu D, Zhuang Z, Wen Z, Zang X, Mo X. MTHFR A1298C polymorphisms reduce the risk of congenital heart defects: a meta-analysis from 16 case-control studies. Ital J Pediatr. 2017;43(1):108. Epub 2017/12/06. doi: 10.1186/s13052-017-0425-1 [pii]. ; PubMed Central PMCID: PMC5715640. 29202788 PMC5715640

[9] Liu PF, Ding B, Zhang JY, Mei XF, Li F, Wu P, et al. Association Between MTHFR C677T Polymorphism and Congenital Heart Disease. Int Heart J. 2020;61(3):553–61. Epub 2020/05/19. doi: 10.1536/ihj.19-389 . 32418960

[10] Cai B, Zhang T, Zhong R, Zou L, Zhu B, Chen W, et al. Genetic variant in MTRR, but not MTR, is associated with risk of congenital heart disease: an integrated meta-analysis. PLoS One. 2014;9(3):e89609. Epub 2014/03/07. doi: 10.1371/journal.pone.0089609 PONE-D-13-26033 [pii]. ; PubMed Central PMCID: PMC3942359. 24595101 PMC3942359

[11] Iacobazzi V, Infantino V, Castegna A, Andria G. Hyperhomocysteinemia: related genetic diseases and congenital defects, abnormal DNA methylation and newborn screening issues. Mol Genet Metab. 2014;113(1–2):27–33. Epub 2014/08/05. doi: 10.1016/j.ymgme.2014.07.016 S1096-7192(14)00220-0 [pii]. . 25087163

[12] Yu D, Yang L, Shen S, Fan C, Zhang W, Mo X. Association between methionine synthase reductase A66G polymorphism and the risk of congenital heart defects: evidence from eight case-control studies. Pediatr Cardiol. 2014;35(7):1091–8. Epub 2014/06/11. doi: 10.1007/s00246-014-0948-9 . 24913415

[13] Matthews RG, Sheppard C, Goulding C. Methylenetetrahydrofolate reductase and methionine synthase: biochemistry and molecular biology. Eur J Pediatr. 1998;157 Suppl 2:S54–9. Epub 1998/05/20. doi: 10.1007/pl00014305 . 9587027

[14] Harmon DL, Shields DC, Woodside JV, McMaster D, Yarnell JW, Young IS, et al. Methionine synthase D919G polymorphism is a significant but modest determinant of circulating homocysteine concentrations. Genet Epidemiol. 1999;17(4):298–309. Epub 1999/10/16. doi: 10.1002/(SICI)1098-2272(199911)17:4&lt;298::AID-GEPI5&gt;3.0.CO;2-V [pii] . 10520212

[15] Zhu WL, Cheng J, Dao JJ, Zhao RB, Yan LY, Li SQ, et al. Polymorphism of methionine synthase gene in nuclear families of congenital heart disease. Biomed Environ Sci. 2004;17(1):57–64. Epub 2004/06/19. . 15202865

[16] Galdieri LC, Arrieta SR, Silva CM, Pedra CA, D’Almeida V. Homocysteine concentrations and molecular analysis in patients with congenital heart defects. Arch Med Res. 2007;38(2):212–8. Epub 2007/01/18. S0188-4409(06)00342-0 [pii] doi: 10.1016/j.arcmed.2006.09.012 . 17227731

[17] Liu YS, Yin XG, Wang JF, Yu LF, Liu HP, Meng FC, et al. [Relationship between genetic polymorphism of homocysteine metabolism enzyme and congenital heart disease]. Chin J Cardiovasc Rev. 2007;5(3):210–3.

[18] Shaw GM, Lu W, Zhu H, Yang W, Briggs FB, Carmichael SL, et al. 118 SNPs of folate-related genes and risks of spina bifida and conotruncal heart defects. BMC Med Genet. 2009;10:49. Epub 2009/06/06. doi: 10.1186/1471-2350-10-49 [pii]. ; PubMed Central PMCID: PMC2700092. 19493349 PMC2700092

[19] Gong T, Li F, Huang H, Dong XY, Feng J, Tang XN. [Relationship of maternal and child polymorphism in methionine synthetase in and methionine synthetase reductase with cogenital heart disease]. Acta Acad Med Mil Tert. 2010;32(2):127–30.

[20] Wang B, Liu M, Yan W, Mao J, Jiang D, Li H, et al. Association of SNPs in genes involved in folate metabolism with the risk of congenital heart disease. J Matern Fetal Neonatal Med. 2013;26(18):1768–77. Epub 2013/05/25. doi: 10.3109/14767058.2013.799648 . 23701284

[21] Mohamad NA, Vasudevan R, Ismail P, Jafar NI, Etemad A, Aziz AF, et al. Analysis of homocysteine metabolism enzyme gene polymorphisms in nonsyndromic congenital heart disease patients among Malaysians. Life Sci J. 2014;11(8):318–26.

[22] Zhao JY, Qiao B, Duan WY, Gong XH, Peng QQ, Jiang SS, et al. Genetic variants reducing MTR gene expression increase the risk of congenital heart disease in Han Chinese populations. Eur Heart J. 2014;35(11):733–42. Epub 2013/06/27. doi: 10.1093/eurheartj/eht221 eht221 [pii]. . 23798577

[23] Shi H, Yang S, Liu Y, Huang P, Lin N, Sun X, et al. Study on Environmental Causes and SNPs of MTHFR, MS and CBS Genes Related to Congenital Heart Disease. PLoS One. 2015;10(6):e0128646. Epub 2015/06/04. doi: 10.1371/journal.pone.0128646 PONE-D-14-52335 [pii]. ; PubMed Central PMCID: PMC4452709. 26035828 PMC4452709

[24] Elizabeth KE, Praveen SL, Preethi NR, Jissa VT, Pillai MR. Folate, vitamin B12, homocysteine and polymorphisms in folate metabolizing genes in children with congenital heart disease and their mothers. Eur J Clin Nutr. 2017;71(12):1437–41. Epub 2017/09/07. doi: 10.1038/ejcn.2017.135 [pii]. . 28876333

[25] Duan S, Li G, Qiu F, Zhao L, Zhao M, Wang L, et al. [Case-control study on the association between four single nucleotide polymorphisms in folate metabolism way and the risk of congenital heart disease]. Wei Sheng Yan Jiu. 2018;47(4):536–42. Epub 2018/08/08. . 30081977

[26] Su J, Li Z. Analysis of MTR and MTRR Gene Polymorphisms in Chinese Patients With Ventricular Septal Defect. Appl Immunohistochem Mol Morphol. 2018;26(10):769–74. Epub 2018/01/03. doi: 10.1097/PAI.0000000000000512 ; PubMed Central PMCID: PMC6250295. 29293099 PMC6250295

[27] Stroup DF, Berlin JA, Morton SC, Olkin I, Williamson GD, Rennie D, et al. Meta-analysis of observational studies in epidemiology: a proposal for reporting. Meta-analysis Of Observational Studies in Epidemiology (MOOSE) group. JAMA. 2000;283(15):2008–12. Epub 2000/05/02. doi: 10.1001/jama.283.15.2008 [pii]. . 10789670

[28] Higgins J, Green S. Cochrane Handbook for Systematic Reviews of Interventions Version 5.1.0. The Cochrane Collaboration. 2011;www.cochranehandbook.org.

[29] Jiang L, Wang K, Lo K, Zhong Y, Yang A, Fang X, et al. Sex-Specific Association of Circulating Ferritin Level and Risk of Type 2 Diabetes: A Dose-Response Meta-Analysis of Prospective Studies. J Clin Endocrinol Metab. 2019;104(10):4539–51. Epub 2019/05/11. doi: 10.1210/jc.2019-00495 [pii]. . 31074789

[30] Xu M, Lin Z. Genetic influences of dopamine transport gene on alcohol dependence: a pooled analysis of 13 studies with 2483 cases and 1753 controls. Prog Neuropsychopharmacol Biol Psychiatry. 2011;35(5):1255–60. Epub 2010/11/17. doi: 10.1016/j.pnpbp.2010.11.001 S0278-5846(10)00414-8 [pii]. ; PubMed Central PMCID: PMC5335908. 21078357 PMC5335908

[31] Xu M, Sham P, Ye Z, Lindpaintner K, He L. A1166C genetic variation of the angiotensin II type I receptor gene and susceptibility to coronary heart disease: collaborative of 53 studies with 20,435 cases and 23,674 controls. Atherosclerosis. 2010;213(1):191–9. Epub 2010/08/25. doi: 10.1016/j.atherosclerosis.2010.07.046 S0021-9150(10)00591-5 [pii]. . 20732682

[32] Xu MQ, Ye Z, Hu FB, He L. Quantitative assessment of the effect of angiotensinogen gene polymorphisms on the risk of coronary heart disease. Circulation. 2007;116(12):1356–66. Epub 2007/09/12. CIRCULATIONAHA.107.728857 [pii] doi: 10.1161/CIRCULATIONAHA.107.728857 . 17846284

[33] Wells GA, Shea B, O’Connell D, Peterson J, Welch V, Losos M, et al. The Newcastle-Ottawa Scale (NOS) for assessing the quality of nonrandomised studies in meta-analyses. 2010;http://www.ohri.ca/programs/clinical_epidemiology/oxford.asp.

[34] Higgins JP, Thompson SG. Quantifying heterogeneity in a meta-analysis. Stat Med. 2002;21(11):1539–58. Epub 2002/07/12. doi: 10.1002/sim.1186 . 12111919

[35] Patsopoulos NA, Evangelou E, Ioannidis JP. Sensitivity of between-study heterogeneity in meta-analysis: proposed metrics and empirical evaluation. Int J Epidemiol. 2008;37(5):1148–57. Epub 2008/04/22. doi: 10.1093/ije/dyn065 [pii]. . 18424475 PMC6281381

[36] Egger M, Davey Smith G, Schneider M, Minder C. Bias in meta-analysis detected by a simple, graphical test. BMJ. 1997;315(7109):629–34. Epub 1997/10/06. doi: 10.1136/bmj.315.7109.629 ; PubMed Central PMCID: PMC2127453. 9310563 PMC2127453

[37] Ouyang S, Li Y, Liu Z, Chang H, Wu J. Association between MTR A2756G and MTRR A66G polymorphisms and maternal risk for neural tube defects: a meta-analysis. Gene. 2013;515(2):308–12. Epub 2012/12/26. doi: 10.1016/j.gene.2012.11.070 S0378-1119(12)01522-3 [pii]. . 23266814

[38] Ouyang S, Liu Z, Li Y, Wu J. Meta-analyses on the association of MTR A2756G and MTRR A66G polymorphisms with neural tube defect risks in Caucasian children. J Matern Fetal Neonatal Med. 2013;26(12):1166–70. Epub 2013/02/22. doi: 10.3109/14767058.2013.777699 . 23425389

[39] Yang M, Yang L, Qi L, Guo Y, Lin X, Zhang Y, et al. Association between the methionine synthase A2756G polymorphism and neural tube defect risk: a meta-analysis. Gene. 2013;520(1):7–13. Epub 2013/02/27. doi: 10.1016/j.gene.2013.02.005 S0378-1119(13)00174-1 [pii]. . 23438943

[40] Yang M, Gong T, Lin X, Qi L, Guo Y, Cao Z, et al. Maternal gene polymorphisms involved in folate metabolism and the risk of having a Down syndrome offspring: a meta-analysis. Mutagenesis. 2013;28(6):661–71. Epub 2013/09/27. doi: 10.1093/mutage/get045 [pii]. . 24068460

[41] Lei W, Xia Y, Wu Y, Fu G, Ren A. Associations Between MTR A2756G, MTRR A66G, and TCN2 C776G Polymorphisms and Risk of Nonsyndromic Cleft Lip With or Without Cleft Palate: A Meta-Analysis. Genet Test Mol Biomarkers. 2018;22(8):465–73. Epub 2018/07/14. doi: 10.1089/gtmb.2018.0037 . 30004262

[42] Mostowska A, Hozyasz KK, Jagodzinski PP. Maternal MTR genotype contributes to the risk of non-syndromic cleft lip and palate in the Polish population. Clin Genet. 2006;69(6):512–7. Epub 2006/05/23. CGE618 [pii] doi: 10.1111/j.1399-0004.2006.00618.x . 16712703

[43] Deng C, Deng Y, Xie L, Yu L, Liu L, Liu H, et al. Genetic polymorphisms in MTR are associated with non-syndromic congenital heart disease from a family-based case-control study in the Chinese population. Sci Rep. 2019;9(1):5065. Epub 2019/03/27. doi: 10.1038/s41598-019-41641-z [pii]. ; PubMed Central PMCID: PMC6433945. 30911047 PMC6433945

[44] Williams K, Carson J, Lo C. Genetics of Congenital Heart Disease. Biomolecules. 2019;9(12). Epub 2020/01/01. E879 [pii] [pii]. doi: 10.3390/biom9120879 ; PubMed Central PMCID: PMC6995556. 31888141 PMC6995556

[45] Nees SN, Chung WK. The genetics of isolated congenital heart disease. Am J Med Genet C Semin Med Genet. 2020;184(1):97–106. Epub 2019/12/27. doi: 10.1002/ajmg.c.31763 ; PubMed Central PMCID: PMC8211463. 31876989 PMC8211463

[46] Jin G, Xu M, Zou M, Duan S. The Processing, Gene Regulation, Biological Functions, and Clinical Relevance of N4-Acetylcytidine on RNA: A Systematic Review. Mol Ther Nucleic Acids. 2020;20:13–24. Epub 2020/03/15. S2162-2531(20)30072-X [pii] doi: 10.1016/j.omtn.2020.01.037 ; PubMed Central PMCID: PMC7068197. 32171170 PMC7068197

[47] Zheng S, Zhao T, Yuan S, Yang L, Ding J, Cui L, et al. Immunodeficiency Promotes Adaptive Alterations of Host Gut Microbiome: An Observational Metagenomic Study in Mice. Front Microbiol. 2019;10:2415. Epub 2019/11/30. doi: 10.3389/fmicb.2019.02415 ; PubMed Central PMCID: PMC6853035. 31781050 PMC6853035

[48] Mandaviya PR, Stolk L, Heil SG. Homocysteine and DNA methylation: a review of animal and human literature. Mol Genet Metab. 2014;113(4):243–52. Epub 2014/12/03. doi: 10.1016/j.ymgme.2014.10.006 S1096-7192(14)00315-1 [pii]. . 25456744

[49] Xia J, Wang Y, Zhang H, Hu Y. Association between MTR A2756G polymorphism and childhood acute lymphoblastic leukemia: a meta-analysis. Leuk Lymphoma. 2014;55(6):1388–93. Epub 2013/08/03. doi: 10.3109/10428194.2013.830304 . 23906019

[50] Yu K, Zhang J, Dou C, Gu S, Xie Y, Mao Y, et al. Methionine synthase A2756G polymorphism and cancer risk: a meta-analysis. Eur J Hum Genet. 2010;18(3):370–8. Epub 2009/10/15. doi: 10.1038/ejhg.2009.131 [pii]. ; PubMed Central PMCID: PMC2987221. 19826453 PMC2987221

[51] Lu M, Wang F, Qiu J. Methionine synthase A2756G polymorphism and breast cancer risk: a meta-analysis involving 18,953 subjects. Breast Cancer Res Treat. 2010;123(1):213–7. Epub 2010/01/30. doi: 10.1007/s10549-010-0755-9 . 20111902

[52] Chowdhury S, Cleves MA, MacLeod SL, James SJ, Zhao W, Hobbs CA. Maternal DNA hypomethylation and congenital heart defects. Birth Defects Res A Clin Mol Teratol. 2011;91(2):69–76. Epub 2011/01/22. doi: 10.1002/bdra.20761 ; PubMed Central PMCID: PMC3168545. 21254366 PMC3168545

[53] Zhang F, Baranova A, Zhou C, Cao H, Chen J, Zhang X, et al. Causal influences of neuroticism on mental health and cardiovascular disease. Hum Genet. 2021;140(9):1267–81. Epub 2021/05/12. doi: 10.1007/s00439-021-02288-x [pii]. . 33973063

[54] Zhang F, Rao S, Cao H, Zhang X, Wang Q, Xu Y, et al. Genetic evidence suggests posttraumatic stress disorder as a subtype of major depressive disorder. J Clin Invest. 2021. Epub 2021/04/28. doi: 10.1172/JCI145942 145942 [pii]. . 33905376 PMC8803333

[55] Wang X, Fang X, Zheng W, Zhou J, Song Z, Xu M, et al. Genetic Support of A Causal Relationship Between Iron Status and Type 2 Diabetes: A Mendelian Randomization Study. J Clin Endocrinol Metab. 2021;106(11):e4641–e51. Epub 2021/06/20. doi: 10.1210/clinem/dgab454 [pii]. ; PubMed Central PMCID: PMC8530720. 34147035 PMC8530720

[56] Liu M, Li F, Yan H, Wang K, Ma Y, Shen L, et al. A multi-model deep convolutional neural network for automatic hippocampus segmentation and classification in Alzheimer’s disease. Neuroimage. 2020;208:116459. Epub 2019/12/15. S1053-8119(19)31050-X [pii] doi: 10.1016/j.neuroimage.2019.116459 . 31837471

